# Bovine trophoblastic cell differentiation and binucleation involves enhanced endogenous retrovirus element expression

**DOI:** 10.1186/1477-7827-10-41

**Published:** 2012-05-25

**Authors:** Katsuo Koshi, Yasunori Suzuki, Yuki Nakaya, Kei Imai, Misa Hosoe, Toru Takahashi, Keiichiro Kizaki, Takayuki Miyazawa, Kazuyoshi Hashizume

**Affiliations:** 1 Laboratory of Veterinary Physiology, Department of Veterinary Medicine, Faculty of Agriculture, Iwate University, 3-18-8 Ueda, Morioka, Iwate, 020-8550, Japan; 2 United Graduate School of Veterinary Science, Gifu University, Gifu, 501-1193, Japan; 3 Tokyo Metropolitan Institute of Public Health, Tokyo, 169-0073, Japan; 4 Laboratory of Signal Transduction, Department of Cell Biology, Institute for Virus Research, Kyoto University, 53 Shogoin-Kawaharacho, Sakyo-ku, Kyoto, 606-8507, Japan; 5 National Livestock Breeding Center, Nishigo-mura, Fukushima, 961-8511, Japan; 6 Department of Developmental Biology, National Institute of Agrobiological Sciences, Ikenodai 2, Tsukuba, Ibaraki, 305-8602, Japan

**Keywords:** Endogenous retrovirus, Trophoblastic cells, Binucleate cells, Matrigel

## Abstract

**Background:**

Endogenous retrovirus (ERV) envelope (env) genes are involved in the differentiation of trophoblastic cells in humans and mice. However, there is limited information about their roles in ruminant trophoblastic cells. Thus, we attempted to explore the possible roles of ERV elements in the binucleation of bovine trophoblastic cells using *in vitro* bovine trophoblastic (BT) cell lines.

**Methods:**

In this study, blastocysts and elongated embryos were obtained from Japanese Black cows, and endometrial and fetal membrane tissues were collected from day 17 to 37 of gestation. The gene expression levels of four ERV elements, *bERVE* (bovine endogenous retrovirus envelope element-like transcript) *-A*, *bERVE–B,* BERV (bovine endogenous retrovirus) -K1 *env*, and BERV-K2 *env*, were analyzed in the fetal and endometrial tissue and cultured BT cell lines using quantitative RT-PCR. On-Matrigel gel and on-collagen gel culturing were used to induce binucleate cell (BNC) formation in the BT cell lines. How the culture conditions affected the expression of BNC-specific genes and ERV elements was examined by quantitative RT-PCR and immunocytochemistry.

**Results:**

*bERVE-A*, *bERVE–B,* BERV-K1 *env*, and BERV-K2 *env* were expressed in almost all BT cell lines; however, only *bERVE-A* and BERV-K1 *env* were detected in trophoblastic tissues during the peri-implantation period. In the on-Matrigel cultures, the expression levels of BNC-specific genes and molecules were enhanced in the BT cells. The expression levels of *bERVE-A* and BERV-K1 *env* were also increased in the BT cells during on-Matrigel culturing. The BT cell expression levels of these ERV elements were consistent with those of BNC-specific genes during on-Matrigel culturing (*P* < 0.01).

**Conclusions:**

These results suggest that *bERVE-A* and BERV-K1 *env* are involved in the expression of BNC-specific genes and the progression of bovine trophoblastic cell binucleation, as their expression levels increased during periods of increased BNC-specific molecule expression, which is strongly suggestive of the development of BNC from mononucleate trophoblastic cells. The on-Matrigel culture system is a convenient *in vitro* tool for studying bovine trophoblastic cell lineages.

## Background

Trophoblastic cells are derived from the outer layer of the morula and can be clearly identified at the blastocyst stage. In addition, trophoblasts, which subsequently develop into the placenta, are the first to differentiate during embryogenesis. There are two major types of bovine trophoblast cells, binucleate cells (BNC) and mononucleate cells (MNC), and they play a crucial role in bovine placentation [1]. BNC display polyploid nuclei and appear in trophoblastic tissues just before implantation. They account for about 20% of trophoblastic cells throughout gestation [2-4]. BNC express various specific genes, such as placental lactogen (*bCSH1*) [5], prolactin-related proteins (PRP) [6], pregnancy-associated glycoproteins (PAG) [7], steroid hormones, prostaglandins, heparanase, etc. [8,9], which are secreted into the maternal circulation. Around implantation, BNC migrate into the endometrial epithelial layer, and some BNC fuse with uterine epithelial cells [2,3,10]. These findings suggest that BNC play a major role in implantation in cows; however, it remains to be elucidated how binucleation is regulated. In rodents, trophoblastic cells include several specialized cell subtypes, including trophoblast giant cells, which are known to display polyploid nuclei and arise by endoreduplication without cytokinesis [11,12]. Syncytiotrophoblasts, which are unique and multi-nucleated cells, develop by fusion induced by endogenous retrovirus (ERV) envelope (env) genes [13,14]. ERV genes have been identified in all vertebrates and are thought to arise from ancient infections of the germ line of the host by exogenous retroviruses. ERV play a pivotal role in placental development, and the ERV envelope genes *syncytin-1* and *syncytin-2* in humans and *syncytin-A* and *syncytin-B* in rodents have been found to display fusogenic activity [13-16]. However, in ruminants the mechanism by which these activities are regulated remains unknown. Recently, it has been proposed that Jaagsiekte sheep retrovirus (enJSRV) is associated with binucleation and/or the properties of BNC, since trophoblastic binucleation was inhibited by the in utero injection of antisense oligonuc-leotides for enJSRV *env*[17,18]. Recently, we found that several ERV elements in the bovine placenta, including *bERVE-A* and BERV-K1 *env*, are predominantly expressed in placental trophoblastic tissues and/or BNC [19,20]. However, the detailed regulatory mechanisms controlling the expression of cell-specific genes and their relevance to binucleation remain unclear. In this study, we explored whether ERV elements participate in the binucleation process in bovine trophoblastic cells using an *in vitro* trophoblastic cell model.

In humans and rodents, there have been many reports about the differentiation of trophoblastic cells in *in vitro* cell cultures [21-29]. Induced human syncytiotrophoblasts displayed upregulated intracellular cyclic AMP expression and markedly increased *syncytin-1* gene expression *in vitro*[28,29]. Although these findings suggest that similar processes could occur in bovine trophoblastic cells, there have been no reports about this issue. We reported that binuc-leation was induced in a bovine trophoblastic cell line (BT-1) cultured on thick collagen gel (collagen I) [30]. Recently, we established twelve bovine trophoblastic cell lines (BT-A to BT-L) from *in vitro* fertilized embryos using bone morphogenetic protein-4 (BMP4) [31]. BT cells are used as a model trophoblastic cell lineage because certain cell culture conditions are known to enhance their differentiation from MNC to BNC [31,32]. The purpose of this study is to examine the expression of ERV elements in bovine trophoblastic cell lines under different *in vitro* cell culture conditions.

## Methods

### Cell culture

BT cell lines (BT-1 and BT-A to BT-L) were established from *in vitro* matured and *in vitro* fertilized blastocysts and cultured, as described previously [31,33]. They were cultured and maintained according to a previously described method [32]. In brief, the cells were cultured in Dulbecco's modified Eagle's medium (DMEM)/F-12 medium (Sigma, Saint Louis, MI, USA) containing 100 IU/ml of penicillin and 100 μg/ml of streptomycin (Sigma) supplemented with 10% fetal bovine serum (FBS; HANA-NESCO, Tokyo, Japan) at 37°C in an atmosphere of 5% CO_2_. The medium was changed every two or three days. A monolayer of confluent BT cells was mechanically passaged by pipetting. Collagen-coated flasks were prepared by incubating a ten-fold diluted solution of acid-soluble porcine type I collagen (3 mg/ml of type I-C collagen; Nitta Gelatin Osaka, Japan) in flasks for more than one hour and then washed with general culture medium. The dissociated cell clumps in the medium were plated in collagen-coated flasks after they had been washed with phosphate-buffered saline (PBS). Bovine cotyledonary fibroblast cells (CF), endometrial fibroblast cells (EF), and epithelial cells (BEE) were derived from cotyledonary and endometrial tissue, respectively, as reported previously [34,35]. In brief, to isolate the CF and EF, small pieces of tissue, which were obtained from the uteri of Japanese Black cattle, were subjected to explant culture, and the cells that grew around the explanted tissue were collected and passaged at least three times to generate a fibroblast cell population. The endometrial epithelium was scraped off from the uterine lumen using a surgical blade and were plated in 6-well microplates coated with type I collagen after being washed several times with DMEM. The phenotypes of the cells were confirmed by immunocytochemical detection with vimentin and/or cytokeratin. They were cultured in DMEM/F-12 containing 100 IU/ml of penicillin and 100 μg/ml of streptomycin supplemented with 10% FBS at 37°C in an atmosphere of 5% CO_2_. The cells were used at the following passage numbers for the examination of ERV derived gene expression in the bovine trophoblastic cell lines: BT-1, around the 300-350th passage; other BT cell lines, around the 30-60th passage; CF, EF, and BEE, around the 5th passage. The cell cultures grown in collagen-coated flasks, on collagen gel (on-collagen cultures), or on Matrigel (on-Matrigel cultures) were used for RNA purification or immunocytochemistry.

### Animals and tissues collection

Gestation status was defined according to the day of gestation (day of AI = day 0 of gestation). Gestational tissues collected during the peri-implantation period; i.e., from days 17–29, were used as day 20 tissues (total n = 4, fetal membranes were collected from one cow on days 17, 18, 19, 21; endometria were collected from three cows on day 20 and one cow on day 29), whereas those collected during the post-implantation period; i.e., from days 31–37, were used as day 35 tissues (total n = 4, fetal membranes were collected from one cow on day 31, two cows on day 35, and one cow on day 37; endometria were collected from two cows on day 35 and two cows on day 37). All tissues were collected from Japanese Black cattle at the abovementioned functional stages of early gestation, except for the day 20 endometria, which were collected from Holstein cows, and subjected to quantitative real-time RT-PCR (qRT-PCR). Blastocysts and elongated embryos were derived from fertilized ova by *in vitro* fertilization, as reported previously [36]. qRT-PCR was performed using blastocysts, elongated embryos, and endometrial and fetal membrane tissues obtained during gestation. All tissues were snap frozen in liquid nitrogen immediately after collection and stored at −80°C until the RNA extraction. All animal procedures were carried out in accordance with the guidelines and ethics set out by the Animal Care and Use Committee of Iwate University and the National Institute of Agrobiological Sciences, Japan.

### RNA extraction and RT-PCR

Total RNA was isolated from the cell cultures and bovine tissues using TRIzol (Invitrogen) according to the manufacturer's instructions. The yield of total RNA was quantified by measuring the absorbance at 260 nm (A260). RNA quality was determined by measuring the A260/A280 ratio using a NanoDrop spectrophotometer (ND-1000, Wilmington, DE, USA) and 1% agarose gel electrophoresis. Genomic DNA was removed using DNase and the Turbo DNA Free Kit (Ambion, Austin, TX, USA). Two micrograms of total RNA were reverse transcribed into cDNA using random primers and a high capacity reverse transcriptase kit (Applied Biosystems, Foster City, CA, USA), according to the manufacturer's instructions. The RT cycle comprised 25°C for 10 minutes, 37°C for 120 minutes, and 85°C for 5 seconds in a thermal cycler, and the resultant cDNA were stored at −20°C. PCR primers that we had designed in previous studies [19,20,31] were used (Table 1). The PCR reaction was performed at 95°C for 30 seconds for denaturing, 60°C for 30 seconds for annealing and 72°C for 1 minute for extension and was repeated for 30 cycles. The PCR products were subjected to electrophoresis using 2% gel and stained with ethidium bromide solution. The amplified products were subcloned into the pGEM-T Easy vector (Promega, Madison, WI, USA) and sequenced using the Big Dye Terminator cycle sequencing kit and an automated sequencer (Applied Biosystems).

**Table 1:** Oligonucleotide primers used for the RT-PCR analysis

**Gene names**	**Accession No.**	**Primers**		**Product**
*GAPDH*	NM_001034034.1	Forward	CCTTCATTGACCTTCACTACATGGTCTA	173 - 200
		Reverse	GCTGTAGCCAAATTCATTGTCGTACCA	1029 - 1003
*bCSH1*	NM_181007.2	Forward	CCATCTCCCCATCAGCAGCAGT	105 - 126
		Reverse	GAGACCCATTACACCCAAACAT	975 - 954
*bPRP-1*	NM_174159.2	Forward	CACGGTCAACAGGAGTCCTCACC	43 - 65
		Reverse	AATTTCAGGTAGCCCGCTGTGG	873 - 852
*bPAG1*	NM_174411.2	Forward	CACCATTGGAACACCCCC	245 - 262
		Reverse	CACTGGGTAGTTGATGCCGTT	989 - 969
*IFNT*	NM_001015511.2	Forward	TCCCCATGGCCTTCGTGCTCTCTCT	75 - 99
		Reverse	CTCAAAGTGAGTTCAGATCTCCACC	668 - 644
*bERVE-A*	NW_001493691.1	Forward	TTCCTCAAAGAAGAAGAGGTAGAACAA	703277-703304
		Reverse	GGGTCCAAATAAGAGGAATAGAATGAT	703748-703775
*bERVE-B*	NW_001493674.2	Forward	GAACTTAATGAGGATATGGAGCAGGTA	3100208-3100181
		Reverse	GACGTTTTGGGTAATCTTTAGTTGAGA	3099456-3099429
BERV-K1 *env*	NW_001495384.1	Forward	GGATGCAATTTGGATCCCAGAC	218834-218855
		Reverse	CCTTTGCATATTAGGCCTCTCCG	221044-221022
BERV-K2 *env*	NW_001494209.3	Forward	TCCACAGGACGCAGATTCTCC	1926147-1926167
		Reverse	CCTTTGCGTATGCCGAGCCTCCT	1928389-1928367

### Quantitative real-time RT-PCR (qRT-PCR)

The expression levels of four ERV elements, *bERVE-AbERVE–B,* BERV-K1 *env*, and BERV-K2 *env*, in the tissues and cultured cells were confirmed by qRT-PCR using the SYBR Green assay (Applied Biosystems), as described previously [20] (Table 2). Several trophoblastic cell lines were confirmed to express *bCSH1, bPRP-1bPAG1*, and interferon tau (*IFNT).* The primer pairs used in the SYBR Green assay were designed in previous reports [19,20,31] (Table 2). The standard curves for each gene were generated by the serial dilution of plasmids containing cDNA for one of these genes in order to quantify their mRNA concentrations. We confirmed the utility of the melting curve for detecting the SYBR Green-based objective amplicon because SYBR Green also detects double-stranded DNA including primer dimers, contaminating DNA, and PCR products from misannealed primers. Contaminating DNA and primer dimers appear as separate peaks from the desired amplicon peak. The expression ratio of each gene to *GAPDH* mRNA was calculated to adjust for variations in the RT-PCR reaction.

**Table 2:** Oligonucleotide primers used for the qRT-PCR analysis

**Gene names**	**Accession No.**	**Primers**		**Product**
*GAPDH*	NM_001034034.1	Forward	AAGGCCATCACCATCTTCCA	280 - 299
		Reverse	CCACCACATACTCAGCACCAGCAT	355 - 332
*bCSH1*	NM_181007.2	Forward	TGCCACACCGAATTCATGAC	447 - 466
		Reverse	AGGGCTTCGTCCTCTGTATTTG	514 - 493
*bPRP-1*	NM_174159.2	Forward	CACGGAGCTGCAGCATATGA	501 - 520
		Reverse	CCTTGTGGCGCTTGATAGGA	558 - 539
*bPAG1*	NM_174411.2	Forward	TCCACTTTCCGGCTTACCAA	375 - 394
		Reverse	CCTTTCATTCTCCCAGATCCAT	436 - 415
*IFNT*	NM_001015511.2	Forward	CTAGGTGCCAGGCAGAACCT	173 - 192
		Reverse	GGGATGAGGAGAGAGTCTGTTCA	232 - 210
*bERVE-A*	NW_001493691.1	Forward	GGATCTGACGGGAGACACAAA	703321-703301
		Reverse	CACCAATCCGGGAATCTTCA	703281-703260
*bERVE-B*	NW_001493674.2	Forward	GGCCCAAGCACTCCTTCAT	3100010-3099992
		Reverse	CGCCCTTTTTCCCATTTCTT	3099973-3099954
BERV-K1 *env*	AB587259.1	Forward	GGAAATCACCGGGATGTCCT	221-240
		Reverse	GGAGAGGAGGCGCTTACCTG	404-385
BERV-K2 *env*	AB587260.1	Forward	AAAGGAGGTCAGGCCGCCTG	221-240
		Reverse	TGGGGGAGGAGGCGCTTACCT	406-386

### Induction of trophoblastic cell differentiation

BT-1, BT-C, and BT-K cells were cultured in collagen-coated, collagen gel-containing, or Matrigel-containing 12-well plates for 8 days for RNA purification. The collagen-coated 12-well plates were prepared by the abovementioned methods. The collagen gel-containing 12-well plates were prepared using type I collagen (3 mg/ml of type I-A collagen; Nitta Gelatin) according to the manufacturer’s instructions. Eight volumes of the collagen solution were mixed with one volume of ten-fold concentrated HEPES buffered physiological salt solution (pH 7.4) and one volume of 0.05 N NaHCO_3_ buffer solution in a conical tube on ice. We dispensed the mixed gel solution into culture dishes and 12-well plates and incubated it at 37°C for 20–30 min, before using it in the cell culture experiments. After the cells had been cultured, 0.04% collagenase (Sigma) was used to dissolve the collagen gel substrate by incubating it for 30 min at 37°C for RNA purification. Matrigel (BD Matrigel™ Basement Membrane Matrix; BD Biosciences, Bedford, MA, USA) containing 12-well plates were prepared using the thin gel method, according to the manufacturer’s instructions; i.e., 50 μl per square centimeter of Matrigel were added to the plates using cooled pipettes whilst the plates were kept on ice. The plates were then incubated at 37°C for 20–30 min and used for cell culturing. After the cells had been cultured, BD Cell Recovery solution (BD Biosciences) was added to dissolve the Matrigel substrate, and the cells were incubated for 1 hour on ice.

### Immunocytochemistry

BT-1, BT-C, and BT-K cells were cultured on collagen-coated or Matrigel-containing (on-Matrigel cultures) cover slips in dishes for 8 days. The collagen-coated and Matrigel-containing dishes were prepared by the abovementioned methods. Anti-bPRP-1 was kindly provided by Dr. L. A. Schuler (University of Wisconsin, Madison). After the cells had proliferated close to confluence, the slips were fixed in 4% paraformaldehyde in 0.1 M PBS (pH 7.4) at 4°C for 15 min. The cell slips were then incubated with anti-bPRP-1 [37,38] (dilution: 1:1000) or normal rabbit serum for 2 hour at room temperature, and after being washed with PBS the slips were incubated with the secon-dary antibody (Alexa Fluor 488 donkey anti-rabbit IgG; Invitrogen) at a dilution of 1:500 in PBS containing 1% BSA, 0.05% NaN_3_, and 0.3% Triton X-100. The slips were then stained with Hoechst 33342 (5 μg/ml, Invitrogen) in secondary antibody solution. Then, the expression of each protein was examined with an ECLIPSE E600 (Nikon, Tokyo, Japan). Pictures of each sample were taken at three randomly chosen locations and used to quantify the number of bPRP-1-positive cells. The mean percentage of bPRP-1-positive cells was calculated from three different experiments.

### Statistical analysis

All values are presented as the mean ± SEM. qRT-PCR was run in duplicate for each sample. Statistical analysis was performed with the JMP software (SAS Institute Inc., Cary, NC, USA) using one-way ANOVA followed by the Tukey-Kramer test. Different letters indicate significant variation among the tissues. To assess the correlations between the expression levels of ERV elements and BNC-specific genes in BT-1 and -C cells during Matrigel culturing, the expression level of each gene was obtained by subtracting the mean gene expression value obtained during collagen-coated culturing from the gene expression value obtained during Matrigel culturing. The values obtained by this method were subjected to correlation analysis. Pearson’s test was used to determine significance. *P* values of less than 0.05 were considered significant in the correlation analysis.

## Results

### Expression profiles of ERV elements in the BT cell lines

**Figure 1:**
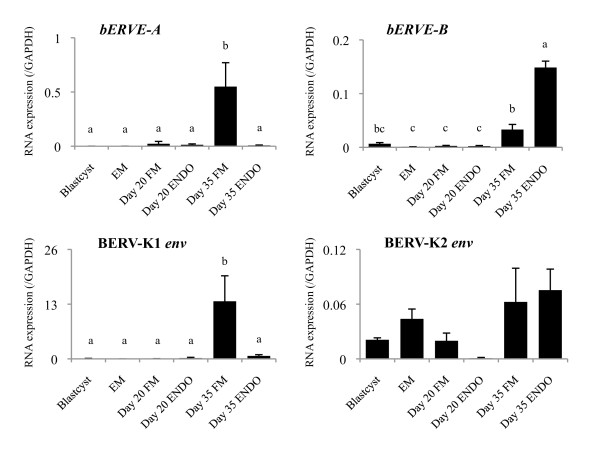
**The gene expression levels of ERV elements during early pregnancy.** Quantitative analysis of the four ERV elements was performed by qRT-PCR, and their expression levels were normalized to *GAPDH* mRNA expression. The bar graph shows mean ± SEM values. Data labeled with different letters are significantly different from each other (*P* < 0.05). EM, elongated embryo; FM, fetal membrane; ENDO, endometrium.

The gene expression levels of ERV elements were re-examined during early pregnancy and used as a control for comparisons with the ERV element expression levels of the *in vitro* cell lines. As a result, very weak *bERVE-A* expression was detected in the embryos at day 20 of gestation, and its expression increased until day 35; however, almost no *bERVE-A* expression was detected in the embryos before day 20 or in the endometrium during the early period of gestation. Although the expression levels of *bERVE-B* were similar to those of *bERVE-A*, it was highly expressed in the endometrium on day 35. BERV-K1 *env* expression was significantly increased in the fetal membranes on day 35 of gestation. The final ERV element, BERV-K2 *env*, was expressed in the embryos on different days as well as in the endometrium (Figure 1).

**Figure 2:**
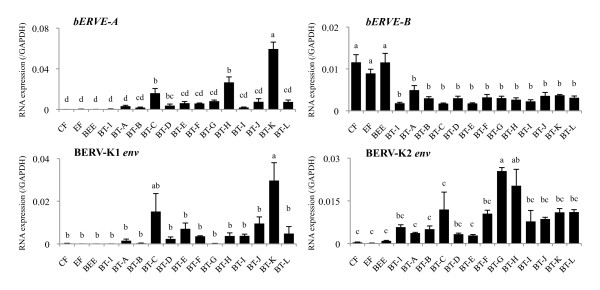
**ERV elements were expressed in the BT cell lines.** The expression levels of four ERV elements, *bERVE-A*, *bERVE–B,* BERV-K1 *env*, and BERV-K2 *env*, in BT cell lines (BT-1, BT-A to BT-L), cotyledonary and endometrial fibroblast cells (CF and EF), and bovine endometrial epithelial cells (BEE) were analyzed by qRT-PCR, and the results were normalized to *GAPDH* mRNA expression. The bar graph shows mean ± SEM values. Data labeled with different letters are significantly different from each other (*P* < 0.05).

The expression levels of the four ERV elements, *bERVE-A*, *bERVE-B*, BERV-K1 *env,* and BERV-K2 *env*, were also examined in 13 BT cell lines (Figure 2). All of the genes were expressed in most of the cell lines (BERV-K1 *env* was not expressed in the BT-1, BT-B, or BT-G cells) but the intensity of their expression depended on the cell line. The BT-C and –K cells displayed higher *bERVE-A* and BERV-K1 *env* expression levels than the other cell lines, especially the BT-K cells (*P* < 0.05). However, the expression levels of the four genes in somatic cells such as CF, EF, and BEE cells were completely different from those observed in the BT cell lines. Almost no expression of *bERVE-A* or BERV-K1 *env* was detected in the somatic cell lines, and much lower expression of BERV-K2 *env* was observed in these cells; however, *bERVE-B* displayed higher expression in these cell lines (*P* < 0.05).

### Induction of binucleation and BNC-specific gene expression during on-matrigel culturing

**Figure 3:**
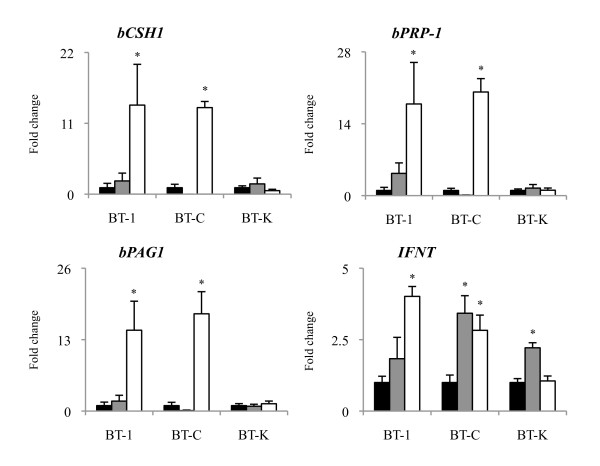
**Induction of BNC-specific genes during the on-Matrigel culturing of BT cell lines.** The BNC-specific gene expression levels of several BT cell lines, including BT-1, BT-C, and BT-K cells, cultured on Matrigel or collagen gel for 8 days. The mRNA expression levels of *bCSH1*, *bPRP-1*, and *bPAG1* (BNC-specific genes) were normalized to *GAPDH* expression, and those of *IFNT* (an MNC-specific gene) were compared with those of the control culture (collagen-coated). The black bars, gray bars, and white bars represent the control, on-collagen gel, and on-Matrigel cultures, respectively. The asterisks indicate a significant difference compared with the control (*P* < 0.05).

**Figure 4:**
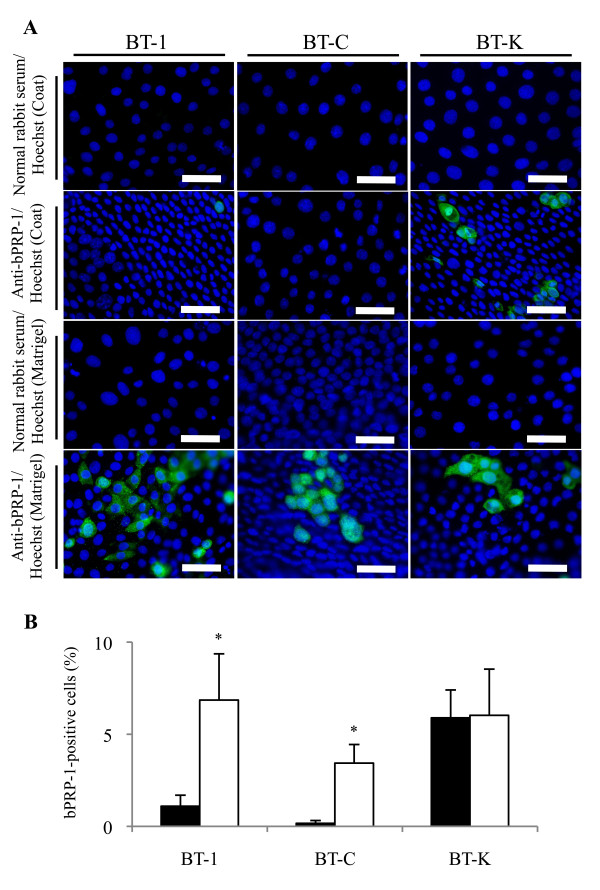
**Induction of bPRP-1 in Matrigel cultured BT cell lines.** (**A**) An immunofluorescent image displaying anti-bPRP-1 staining of BT-1, BT-C, and BT-K cells, which were cultured in collagen-coated wells or by on-Matrigel culturing for 8 days. Nuclear DNA was stained with Hoechst 33342. Bar = 50 μm. (**B**) The percentages of bPRP-1-positive cells in the collagen-coated (black bar) and Matrigel cultures (white bar). Three pictures were taken of random locations in each sample. The mean percentage of bPRP-1-positive cells was calculated from three different experiments. The bar graph shows mean ± SEM values. The asterisks indicate a significant difference compared with the control (*P* < 0.05).

The induction of BNC from MNC in BT cell lines was examined using on-Matrigel cultures. For this purpose, BT-1, BT-C, and BT-K cells were selected, and the expression levels of cell-specific genes were determined: *bCSH1*, *bPRP-1*, and *bPAG1* were used as BNC markers, and *IFNT* was used as an MNC indicator. The expression levels of BNC-specific genes; i.e., *bCSH1*, *bPRP-1*, and *bPAG1*, were significantly increased in the BT-1 and BT-C cells in the 8-day on-Matrigel cultures, while the expression levels of BNC-specific genes were not changed in the BT-K cells. The expression of *IFNT* was significantly increased in the BT-C and BT-K cells grown by on-collagen gel culturing (*P* < 0.05) and the BT-1 and BT-C cells grown by on-Matrigel culturing (Figure 3). In this induction study, we used bPRP-1 as an immunohistochemical marker of binucleation. Compared to the collagen-coated cells, the numbers of bPRP-1-positive cells were significantly increased in the BT-1 and BT-C cells, but not in the BT-K cells grown by on-Matrigel culturing (Figure 4). These results were consistent with the *bPRP-1* expression results obtained by qRT-PCR (Figure 3).

### Induction of the expression of ERV elements in BT cell lines grown by on-matrigel culturing

**Figure 5:**
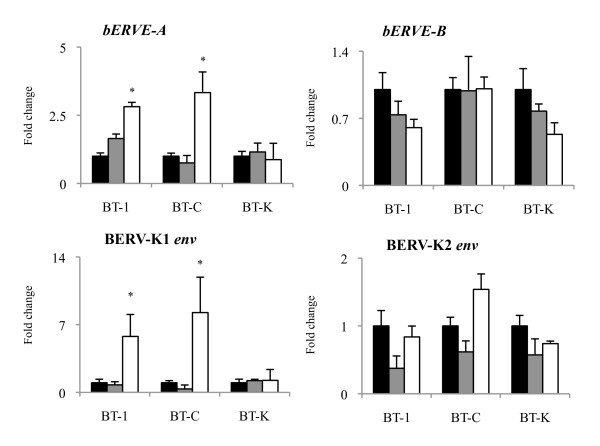
**Induction of ERV element expression during the on-Matrigel culturing of BT cell lines.** The induction of four ERV elements in BT-1, BT-C, and BT-K cells during 8 days on-Matrigel culturing. The data were normalized to the control and then averaged (mean ± SEM). The black bars, gray bars, and white bars represent the control, on-collagen gel, and on-Matrigel cultures, respectively. The asterisks indicate a significant difference compared with the control (*P* < 0.05).

The expression levels of *bERVE-A* and BERV-K1 *env* were significantly increased in the BT-1 and BT-C cells (*P* < 0.05); however, the expression levels of *bERVE-B* and BERV-K2 *env* were not significantly different in any of the cell lines used. The expression levels of the *bERVE-A* and BERV-K1 *env* genes were not changed in the BT-K cells (Figure 5). Since indicators of binucleation, including the upregulation of BNC-specific genes and an increased number of bPRP-1-positive cells, were only detected in the BT-1 and BT-C cells during on-Matrigel culturing (Figure 3, 4), we used BT-1 and BT-C cells to analyze the correlation between binucleation and the expression levels of ERV elements. On-Matrigel culturing enhanced the expression levels of BNC-specific genes; i.e., *bCSH1*, *bPRP-1*, and *bPAG1* and ERV elements, and significant correlations were detected between the expression levels of BNC-specific genes and those of *bERVE-A* and BERV-K1 *env* (Table 3; *P* < 0.01).

**Table 3:** Correlations between the expression levels of ERV elements and BNC-specific genes in BT-1 and BT-C cells during on-Matrigel culturing

				**Correlation coefficients (*r*)**			
	***bPRP-1***	***bPAG1***	***IFNT***	***bERVE-A***	***bERVE-B***	**BERV-K1*env***	**BERV-K2*env***
*bCSH1*	0.94 **	0.92 **	−0.48	0.94 **	0.26	0.92 **	0.59
*bPRP-1*		0.97 **	−0.73	0.91 **	0.46	0.99 **	0.61
*bPAG1*			−0.70	0.94 **	0.34	0.94 **	0.57
*IFNT*				−0.58	−0.69	−0.76*	−0.48
*bERVE-A*					0.22	0.89 **	0.62
*bERVE-B*						0.50	0.71
BERV-K1 *env*							0.59
BERV-K2 *env*							

*: *P* < 0.05, **: *P* < 0.01. The on-Matrigel culture was repeated three times, and the data were compared by examining the fold changes in the expression levels of the genes between the collagen-coated culture and on-Matrigel culture experiments.

## Discussion

The purpose of this study is to examine whether ERV elements are involved in the bovine trophoblastic cell differentiation pathway; i.e., in their differentiation from MNC to BNC/multinuclear cells. We analyzed the following three topics: first, we examined when bovine trophoblastic cells start expressing ERV elements during early embryonic development *in vivo*. Second, we examined whether *in vitro* trophoblastic cell lines are useful for analyzing cell differentiation. Third, we investigated the correlation between ERV element expression and trophoblastic binucleation.

First, the expression profiles of the ERV elements suggested that two genes, *bERVE-A* and BERV-K1 *env*, start to be expressed in trophoblastic tissues on day 20 of gestation and that their expression levels are markedly increased on day 35 of gestation. These two ERV elements are specifically expressed in the fetal membrane on day 35 of gestation, but the other genes, *bERVE-B* and BERV-K2 *env*, are not specifically expressed in the fetal membrane. In addition, *bERVE-A* and BERV-K1 *env* display ERV features. The former has a similar sequence to those of gamma-retroviruses or syncytin-like ERV, and the latter is a beta-retrovirus, the same as enJSRV [19,20,39]. Although their functions have not been clarified, our findings suggest that bovine trophoblastic tissues express two ERV elements with different origins. The ERV element expression levels observed in trophoblastic cells in this study agree with those described in previous reports [19,20] and BNC-specific studies [35,37]. Also, BERV-K1 *env* has been found to perform a specific function in non-trophoblastic cell lines, as has been found for enJSRV [unpublished data; Nakaya Y, Koshi K, Baba K, Nakagawa S, Kizaki K, Hashizume K, Miyazawa T, 2011]. Consequently, these ERV elements might be crucial for trophoblastic cell differentiation because they are expressed during placentomal villus formation [37,40]. These *in vivo* expression data suggest that it is worthwhile exploring the relationship between binucleation and ERV functions.

We have developed various bovine trophoblastic cell lines [31,33] because they are a useful tool for analyzing trophoblastic cell functions and lineages. The BT-1 cell line was established about 10 years ago and is able to differentiate on thick collagen-gel cultures [30]. In the present study, we used the on-Matrigel culture method, which is a more efficient way of inducing BNC formation than on-collagen gel culturing [32,33]. The detailed mechanisms responsible for the binucleation observed during the on-collagen gel and on-Matrigel culturing have not been elucidated; however, improvements in these methods will hopefully lead to the development of BT cell lines that can be used as models for analyzing trophoblastic cell lineages.

The expression profiles of the four ERV elements were unique, and all of the elements were detected in most cell lines, but not in the somatic cell lines (CF, EF, and BEE), except *bERVE-B*, which was expressed in the somatic cell lines. These expression profiles were consistent with those described in previous reports [19,20]; namely, *bERVE-B* was expressed in the endometrium, whereas almost no BERV-K1 *env* expression was detected in the endometrium. Rather intense expression of *bERVE-A* and BERV-K1 *env* was detected in the BT-C and BT-K cells, respectively. These two cell lines strongly express *bCSH1bPRP-1*, and *bPAG1*[31]. Therefore, these results suggest that *bERVE-A* and BERV-K1 *env* might be associated with the fundamental characteristics of BNC, as has been found for other BNC-specific genes. The involvement of ERV elements in bovine trophoblastic cell differentiation was also examined. Most of the BT cell lines expressed ERV elements, especially *bERVE-A* and BREV-K1 *env*. The expression levels of these two elements were enhanced during the induction of BNC by *in vitro* on-Matrigel culturing. These results agree with our hypothesis that ERV elements are involved in the binucleation of bovine trophoblastic cell lineages. The correlations between the expression levels of BNC-specific genes and ERV elements were significantly positive. Why does on-Matrigel culturing enhance the formation of BNC and the expression of ERV elements compared with on-collagen gel culturing and other conventional culture methods? Extracellular matrix remodeling was observed in the bovine fetal membrane and placentome during gestation; in particular, collagen type I became undetectable in the fetal stroma of the placentome [41,42], and *in vitro* studies have suggested that the differentiation of human villous cytotrophoblasts into syncytiotrophoblasts is modulated by several cytokines [43]. Thus, in addition to collagen gel, we made use of Matrigel, which is known to contain basal membrane components and abundant cytokines, to examine the expression of ERV elements in bovine trophoblastic cell lines under different *in vitro* cell culture conditions in this study. In our previous study, we used on-collagen gel culturing and detected increased BNC development after 2 weeks, and we also found that β-catenin might play a pivotal role in the binucleation process; however, we have not clarified the detailed mechanism responsible for its effects [44]. On-Matrigel culturing seems to be a more efficient and convenient method for inducing BNC. This might be due to differences in the components of the matrices involved; i.e., Matrigel contains various matrix molecules, mainly laminin, collagen IV, and proteoglycans, because it is extracted from Engelbreth-Holm-Swarm (EHS) murine sarcoma cells. Laminin might be responsible for the increased numbers of BNC produced by on-Matrigel culturing; i.e., BNC produce laminin [42], and the matrix might supply biogenic activity to trophoblastic cells. Another possible explanation is that Matrigel contains matrix degrading enzymes, their inhibitors, and growth factors. FGF2, which is one of the cytokines contained in Matrigel, is also known to be produced by immature BNC throughout gestation [45]. Therefore, FGF2 might be involved in the differentiation of BNC observed in this study. On the other hand, although the addition of FGF to an *in vitro* bovine trophoblastic cell culture resulted in the upregulation of *IFNT* expression [46,47], significantly upregulated *IFNT* expression was detected in the BT-C and BT-K cells after 8 days of on-collagen gel culturing, and a particularly significant increase in IFNT expression was seen in the BT-K cells during on-Matrigel culturing (Figure 3). In the BT-K cells, these cytokines might make a small contribution to the differentiation of MNC compared with extracellular matrix molecules. On the other hand, significantly upregulated *IFNT* expression was detected in the BT-1 and BT-C cells after 8 days on-Matrigel culturing. The effects of these cytokines and extracellular matrix molecules on BT cell lines might depend on the cell lineage; i.e., these BT cell lines include different stages and/or proportions of MNC stem cells. Although BT-K cells strongly express BNC-specific genes, as do BT-C cells [31], these genes were not induced during the on-Matrigel culturing. BT-K cells might include a greater initial number of BNC and hence not need to produce new BNC, whereas BT-C cells might include stem cells and/or be immature because they strongly express *POU5F1*[31].

Although there is no direct evidence for this, these results suggest that bovine ERV elements induce cell fusion, similar to human and rodent syncytins [13-16]. BERV-K1 env might induce such activity, whereas bERVE-A might not, because the former contains a TM domain, but no TM domain has been detected in the latter element. Thus, the TM domain might be a pivotal factor for cell fusion [48]. However, the expression profiles of the abovementioned ERV elements are quite similar, and our statistical analysis suggested that there are significant correlations between the expression levels of *bERVE-A* and/or BERVE-K1 *env* and BNC-specific genes in BT-1 and BT-C cells during on-Matrigel culturing, which induced binucleation. There are many aspects of BNC formation that remain to be examined.

## Conclusions

In conclusion, all of the examined BT cell lines expressed ERV elements, and their expression levels were closely correlated with those of BNC-specific genes and the binuc-leation of bovine trophoblastic cells. On-Matrigel culturing is a convenient *in vitro* tool for studying bovine trophoblastic cell lineages using BT cell lines. ERV elements might be crucial for bovine trophoblastic cell fusion, similar to syncytins in human and rodents.

## Competing interests

The authors declare that they have no competing interests.

## Authors’ contributions

KK designed and performed the whole study and prepared the manuscript. YS performed the BNC induction study involving the on-Matrigel culturing of BT cell lines. KI prepared the bovine embryos. TT and MH collected the bovine tissue samples. YN and TM participated in coordinating the design of the study. Ke K participated in the design and coordination of the study and helped to draft the manuscript. KH participated in the design and coordination of the whole study and helped to draft the manuscript. All the authors have read and approved the final manuscript for publication.
